# Effect of intraoperative hand-grip position on surgical outcome of thumb carpometacarpal arthrodesis

**DOI:** 10.1186/s13018-023-04423-y

**Published:** 2023-12-07

**Authors:** Kai-Xing Alvin Lee, I.-Ting Chung, Wei-Chih Wang, Chen-Wei Yeh, Tsung-Yu Ho, Cheng-En Hsu, Yung-Cheng Chiu

**Affiliations:** 1 School of Medicine, China Medical University, Taichung, 404 Taiwan; 2 Department of Orthopaedics Surgery, China Medical University Hospital, No. 91 Hsueh-Shih Road, Taichung, 404 Taiwan; 3 Department of Orthopaedics Surgery, China Medical University Hsinchu Hospital, Hsinchu, 302 Taiwan; 4 Sports Recreation and Health Management Continuing Studies-Bachelor’s Degree Completion Program, Tunghai University, Taichung, 407 Taiwan; 5 Department of Orthopaedics Surgery, Taichung Veterans General Hospital, Taichung, 407 Taiwan

**Keywords:** Thumb carpometacarpal joint, Osteoarthritis, Arthrodesis, T-hook plate

## Abstract

**Background:**

A variety of surgical techniques had been developed over the past few decades for treating thumb carpometacarpal joint (CMCJ) osteoarthritis (OA). However, there are currently no accepted consensus on the ideal treatment for thumb CMCJ OA. Arthrodesis was one of the widely popular treatment methods; however, studies have showed that non-union rates were as high as 50%, with higher complications such as osteoarthritis of neighbouring joints and higher revision surgeries required as compared to other surgical methods. Patients with arthrodesis were also reported to have decreased thumb range of motion and loss of opponens function. Currently, there are numerous intraoperative positioning techniques for arthrodesis which could be confusing for young surgeons. With recent developments of fixation plates and better understanding of the wrist anatomy, this retrospective review aimed to evaluate the efficacy of our intraoperative hand-grip positioning method for arthrodesis of thumb CMCJ OA. What are the postoperative functional outcomes of (1) T-hook plates and (2) our intraoperative hand-grip positioning method for Eaton III thumb CMCJ OA arthrodesis by evaluating pain visual analogue scale (VAS) score, Disabilities of the Arm, Shoulder and Hand questionnaires (DASH), Mayo Wrist scores, capability of thumb opposition (Kapandji score), and comparing pre- and postoperative grip and pinch strength?

**Methods:**

Twenty patients with CMCJ OA underwent arthrodesis using our intraoperative hand-grip positioning method and T-hook plates and screws (Acumed, USA). Patients were evaluated preoperatively and at 1, 3, 6 and 12 months postoperatively. Radiologic assessment including fusion evaluation, evaluation of radial and palmar abduction angles was done on hand X-rays.

**Results:**

Twenty patients with a minimum follow-up duration of 12 months were included in this study. 100% fusion rate was achieved with only 1 case of complication involving radial sensory nerve neuropathy which was resolved after removal of implant and neurolysis. Significant improvement in pain and Mayo Wrist scores were noted 3 months postoperatively, whilst DASH score exhibited significant improvements after 6 months of follow-up (*p* < 0.05). Even though there were no significant differences observed between preoperative and postoperative grip strength, pinch strength and Kapandji scores, positive recovery trends were noted for all parameters with these functions surpassing preoperative levels after 12 months of follow-up. Significant improvements on hand X-rays were also noted for both postoperative radial and palmar abduction angles.

**Conclusions:**

There is currently no consensus on the ideal treatment method for thumb CMCJ OA. In this study, we would like to propose a simple intraoperative hand-grip positioning method with T-hook plates for arthrodesis. As seen from our results, our technique was able to provide satisfactory and replicable postoperative results and thus we would like to propose our hand-grip positioning method with T-hook plates fixation for subsequent treatment of patients with Eaton stage III thumb CMCJ OA.

## Introduction

Radiographic findings of thumb carpometacarpal joint (CMCJ) osteoarthritis (OA) are commonly found in postmenopausal women, of which more than half of them experienced symptoms such as pain and decreased range of motion in the affected hand [1, 2]. In the 1950s, trapeziectomy was the preferred technique due to its predictable postoperative pain relief results [3–6]. However, more often than not, trapeziectomy was often related to a reduction or loss in gripping strength due to the metacarpal shifting into the trapezium void [7, 8]. Thus, trapeziectomy is now performed in conjunction with ligament reconstruction and tendon interposition (LRTI), using the flexor carpi radialis, abductor pollicis longus, or palmaris longus to fill the void left by the removed trapezium. A systematic review by Martou et al. in 2004 concluded that despite the majority of the review articles recommended trapeziectomy with LRTI as the ideal treatment option for thumb CMCJ OA, many of these studies exhibited methodological flaws and limitations, which consequently diminished the reliability of their findings [8]. A recent systematic review by Wajon et al. reported that there were no superior surgical procedures for thumb CMCJ OA in terms of pain reduction, range of motion and strength improvements [9, 10]. Furthermore, trapeziectomy with LRTI has also been associated with an increased incidence of complications, such as scarring, tenderness, tendon rupture, and sensory loss, compared to other approaches. As a result of the distinct advantages and potential risks associated with each procedure, it has been proposed that the ideal treatment options should be individually tailored based on patient-specific parameters, such as occupation, age, bone quality, and the surgeon’s experience [11]. Current recommendations suggest that arthrodesis should be reserved for younger, active individuals who require a strong pinch strength with an average range of motion. Arthrodesis of thumb CMCJ was first proposed and performed by Muller in 1949. Since then, the technique has gradually evolved in an effort to create as solid a bony bridge as possible [12]. Arthrodesis has been reported to maintain thumb length and pinch strength, however, some surgeons have concern about accepting it as the first-line treatment due to high incidences of loss of opponens function, which is a crucial function for human beings [10, 12]. Furthermore, there was a wide variability in literature reports regarding union rates, ranging from 50 to 100% [13]. Recently, due to improved understanding of bone anatomy, biology, surgical techniques, and implant designs has led to advancements in the field. Some studies indicate impressive outcomes: Muller and Leach achieved a 100% fusion rate using impacted bone grafts, Stark came close with a 95% fusion rate using K-wires, and Pardini achieved a 100% fusion rate utilizing tension band fixation [14–16]. All cases reported pain relief, improved functions and pinch strength. On the other hand, Clough et al. achieved a fusion rate of only 50%; however, patient satisfaction was at 100%, and pinch strength reached 90% at 1 year of follow-up [17]. In the selection of proper fusion plate size, Hanel applied 2.0-mm and 2.7-mm plates on 30 patients with arthrodesis and 100% fusions were achieved [18]. In most of the studies, there was consistent emphasis on the importance of proper metacarpal positioning, which is vital for postoperative opponens function and ultimately impacts patient satisfaction. It was proposed that the thumb is placed 45 degrees to fthe coronal and sagittal planes of the hand with slight pronation to improve postoperative opposition [19]. More often than not, the experience of the hand surgeon is the key factor in ensuring both the quality of fusion and the functional recovery of the thumb.

In our study, we conducted a retrospective review of 20 patients who had undergone arthrodesis for thumb CMCJ OA between the period of January 2019 and December 2022. T-hook plates with 2.3 mm screws (Acumed, USA) were used for arthrodesis and pain visual analogue scale (VAS) score, functional outcomes (Disabilities of the Arm, Shoulder and Hand [DASH] questionnaires, Mayo Wrist scores and Kapandji scores), grip and pinch strength, and complications were evaluated at 1, 3, 6 and 12 months postoperatively. Radiographic assessments, including evaluations of fusion as well as postoperative radial and palmar abduction angles, were performed using hand X-rays. In this study, we introduced an optimal intraoperative hand-grip positioning method that eliminates the need for additional intraoperative fluoroscopy while achieving the ideal fusion angle for the thumb CMCJ. Our technique is simple to execute, even for less-experienced surgeons and it provides replicable and reliable surgical results. A 100% successful fusion rate was achieved, with only 1 complication involving radial sensory nerve neuropathy. This issue was resolved through a second surgery with the removal of the implant and subsequent neurolysis. Significant improvement in pain and Mayo Wrist scores were noted 3 months postoperatively, whilst DASH scores exhibited significant improvements after a 6-month follow-up (*p* < 0.05). Even though there were no significant differences observed between preoperative and postoperative grip strength, pinch strength and Kapandji scores, positive recovery trends were noted for all parameters with these functions exceeding preoperative levels after 12 months of follow-up. Significant improvements were also noted for both postoperative radial and palmar abduction angles on hand X-ray. Our results showed that arthrodesis with our intraoperative hand-grip positioning method was simple to execute and yet was able to provide satisfactory clinical results. Therefore, we would like to propose and recommend our intraoperative hand-grip positioning method for arthrodesis of patients diagnosed with Eaton stage III thumb CMCJ OA.

## Materials and methods

A retrospective review was performed on 20 patients who had undergone arthrodesis for thumb CMCJ osteoarthritis (OA) between January 2019 and December 2022. All patients provided informed consent, and all procedures and recruitment efforts were conducted in accordance with our Institutional Review Board (IRB) protocols (IRB: CMUH111-REC2-132). The main indication for surgery was pain located at the base of the first metacarpal, accompanied by weakness in pinch power. The inclusion criteria for arthrodesis were active patients with radiographic-proven thumb CMCJ OA of Eaton stage III in which conservative treatments had provided limited symptomatic relief. All surgeries were operated on by a single senior hand surgeon (Yung-Cheng, Chiu). Patients with degenerative diseases other than osteoarthritis, had ongoing infections, previous failed surgery required arthrodesis as a salvage procedure, inflammatory arthritis were excluded from this study. In addition, patients above the age of 65 years old and those below the age of 18 were also excluded.

All patients underwent an arthrodesis using a T-hook plate with 2.3 mm screws (Acumed, USA). In all patients, pre- and postoperative functional results were measured using pinch strength, grip power, capability of thumb opposition (Kapandji), DASH and Mayo Wrist scores. Pain score (VAS) was also recorded. Postoperative radiological images were obtained at 1, 3, 6 and 12 months to observe for signs of fusion, evaluation of osteoarthritis and complications of neighbouring joints. Fusion criteria were determined to be pain-free thumb CMCJ when pinching, gripping and presence of bony consolidation and bony bridge in anteroposterior and oblique views of hand X-ray exam. Thumb radial abduction and palmar abduction angle were calculated from the hand anteroposterior view and the hand oblique view respectively. The intercarpal angle was calculated by measuring the angle between the 1st and the 2nd metacarpal by passing a line through the longitudinal axis of both metacarpals.

### Operative techniques

**Fig. 1:**
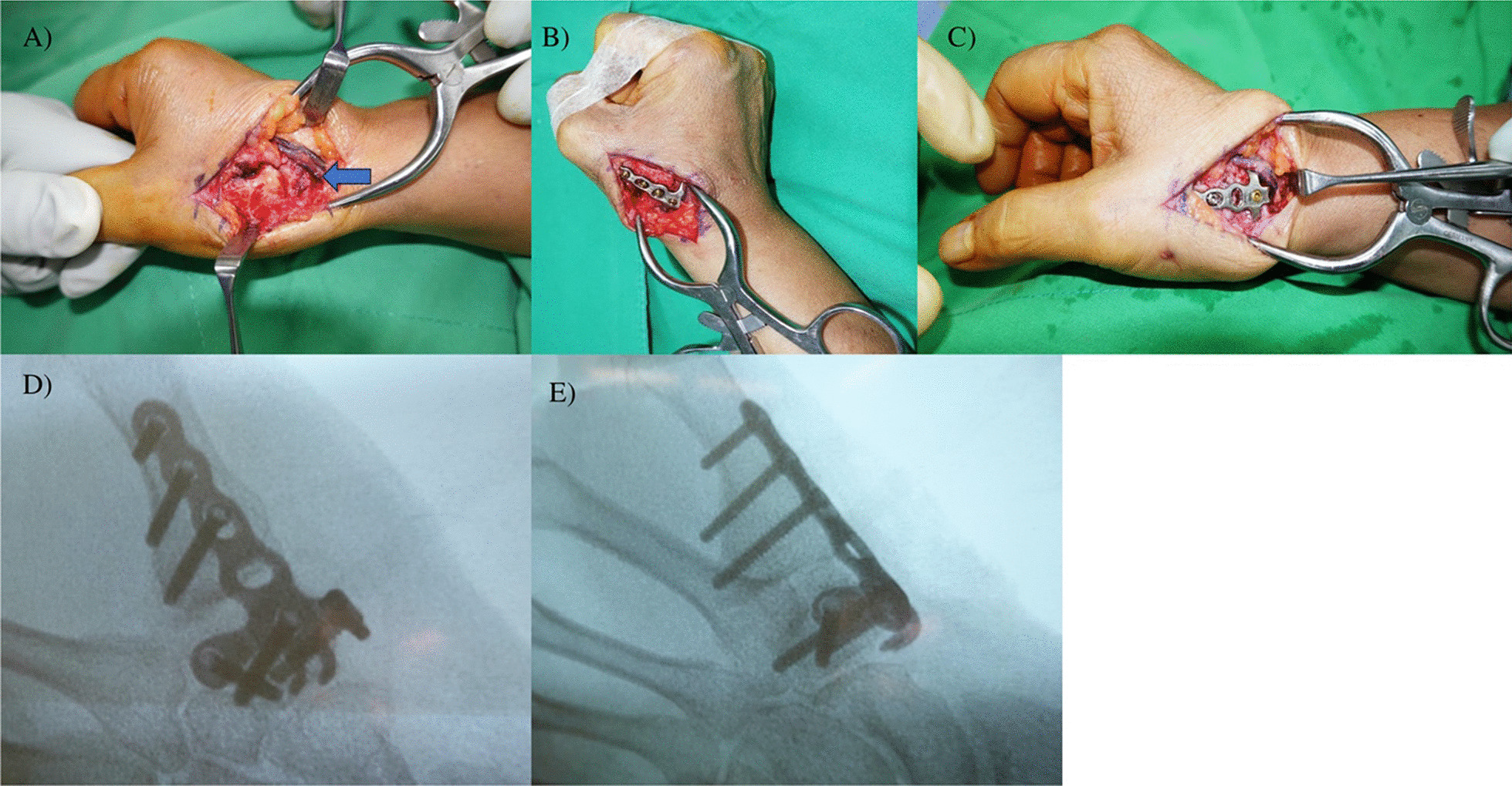
**A** Surgical approach: dorsal incision with neurovascular protection; **B** Our intraoperative hand-grip positioning method with sterile tape fixation; **C** T- hook plate fixation on CMC joint; **D** and **E** Intraoperative fluoroscopy: anteroposterior and lateral views Legend: Arrowhead: Dorsal Radial Artery

A longitudinal linear incision was made on the dorsal side of the thumb's CMC joint, with care taken to protect the dorsal radial artery and radial sensory nerves (Fig. 1a). The extensor pollicis brevis and extensor pollicis longus tendons was retracted to either side. The dorsal joint capsule of the thumb CMC joint was then incised. Osteophytes and synovium were removed, and decortication was performed using a burr to remove the articular cartilage and to expose the cancellous bone. Using sterile adhesive tape, the thumb tip was adhered and aligned with the PIPJ of the middle finger in hand-grip position to ensure that the fusion angle was sufficient to allow opponens function. (Fig. 1b). This positioning method was kept throughout the entire duration of arthrodesis. Subsequently, a 1.2 mm K-wire was utilized to temporarily stabilize the thumb CMC joint. The T-hook plate (Acumed, USA) was then applied for the thumb CMCJ arhrodesis. 3 proximal uni-cortical screws were applied in a convergence manner for trapezium fixation. Then, 3 distal bi-cortical screws were applied for 1^st^ metacarpal shaft fixation (Fig. 1c). The position was confirmed under C-arm guidance, then the K-wire and sterile adhesive tape was removed (Fig. 1d and e). The subcutaneous tissues and skin were subsequently closed in layers, followed by the application of short arm thumb spica protection. Intraoperatively, we would switch to trapeziectomy with LRTI if the trapezium was found to have bone quality too poor to be fixed by T-hook plates.

### Postoperative care

The sutures were removed after 2 weeks of short arm thumb spica protection. Subsequently, patients were advised to continue using thumb spica protection for a duration of 4 weeks. Following this, an active range of motion (ROM) regimen was initiated with wrist brace protection for an additional 4 weeks, whilst simultaneously avoiding forceful motions. Patients were then permitted to gradually resume their normal lifestyle activities after this rehabilitation program.

### Statistical analysis

Data analysis was performed using the SPSS software (Version 20.0; Chicago; Illinois). Student’s t test was used to compare pre- and postoperative scores of pain score (VAS), pinch strength, grip power, Kapandji score, DASH score and Mayo Wrist scores at 3, 6 and 12 months of follow-ups. Furthermore, Student’s t test was also used to compare preoperative and postoperative radial and palmer abduction angles.

A value of p < 0.05 was considered significant.

## Results

**Fig. 2:**
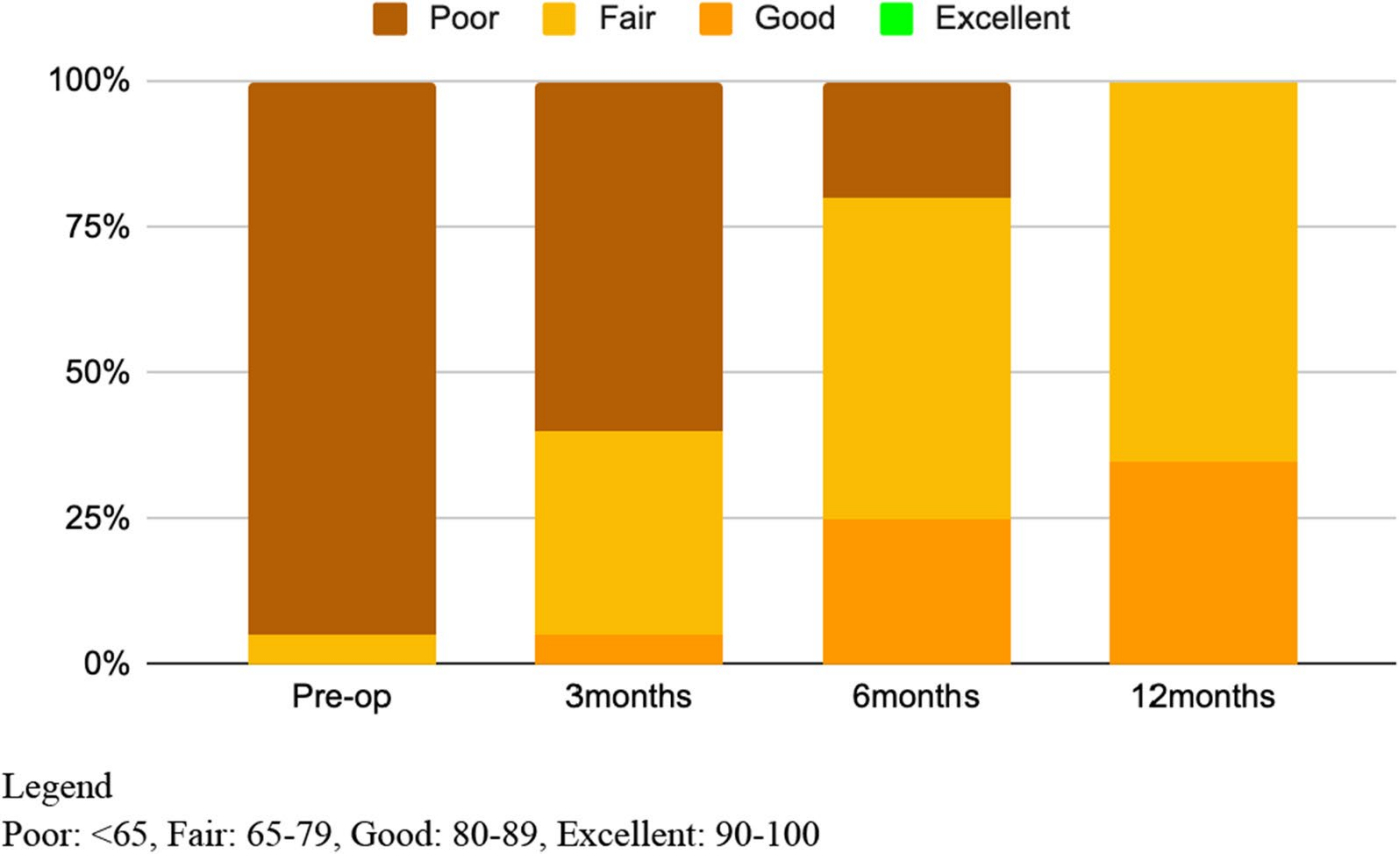
Mayo Wrist Scores Legend Poor: < 65, Fair: 65–79, Good: 80–89, Excellent: 90–100

A total of 20 thumb CMCJ OA patients with a minimum follow-up of 12 months were enrolled in our study (Table 1). Arthrodesis was performed on all patients using our intraoperative hand-grip positioning method. All 20 patients underwent surgery on a single thumb, resulting in a total of 20 thumbs. Among the patients, 82.3% were female, with an average age of 45 years old with 90% of them having right hand as their dominant hand. Additionally, 75% of the surgical procedures were carried out on the left side. In the cohort of 20 patients, 1 case experienced complications involving radial sensory nerve neuropathy. Nevertheless, this issue was resolved 6 months postoperatively after the second surgery with the removal of implant and neurolysis. We compiled preoperative and postoperative follow-up data for the 20 patients, which encompassed measurements of gripping strength, pinching strength, DASH scores, Kapandji scores, pain VAS score, Mayo Wrist scores and radial and palmar abduction angles (Table 2). Notably, no significant differences were observed for preoperative and postoperative grip strength, pinch strength, and Kapandji scores at 3, 6, and 12 months. However, DASH scores exhibited significant improvement (P < 0.05) at 6 and 12 months, while pain VAS scores demonstrated significant enhancement (P < 0.05) at 3, 6, and 12 months, respectively. Preoperative pain VAS score was 6.05 ± 2.31 and subsequent postoperative pain VAS score was 3.75 ± 2.38, 2.15 ± 1.46 and 1.10 ± 1.12 at 3, 6 and 12 months of follow-ups. Mayo Wrist Scores were also tabulated and it could be noted there were statistically significant improvements after 3 months of follow-ups (p < 0.05). Preoperative Mayo Wrist Scores were reported to be 42.00 ± 12.07 with subsequent 55.00 ± 13.57, 68.00 ± 8.34 and 74.75 ± 5.95 at 3, 6 and 12 months of follow-ups, respectively. As seen in Fig. 2, majority of the patients were noted to have poor preoperative Mayo Wrist scores. However, clinical improvements could be gradually noted after arthrodesis and at 12 months of follow-ups, 70% of the patients were noted to have fair functional wrist scores with the other 30% in the good category of the Mayo Wrist scores.

**Table 1:** Demographics and baseline characteristics

	Number of patients (*n* = 20)	%
Thumbs	20	100
Single-sided operations	20	100
Gender		
Male	3	17.7
Female	17	82.3
Age at treatment	45 years old (IQR: 35 to 55)	
Operated hand		
Left	16	75
Right	4	25
Dominant hand		
Left	2	10
Right	18	90
Complications	1 (Sensory deficit)	5
Secondary surgery	1 (Removal of implant)	
Follow up durations	18 months (IQR: 12 to 24)

**Table 2:** Preoperative and postoperative comparison of functional scores and radiographic results (*p* < 0.05)

	Preoperative	Postoperative (3 months)	P value	Postoperative (6 months)	P value	Postoperative (12 months)	P value
*Functional score*							
Grip	16.61 ± 9.28	15.01 ± 9.51	0.27	18.42 ± 9.52	0.19	19.21 ± 9.43	0.09
Pinch	3.72 ± 2.26	3.21 ± 1.76	0.24	3.90 ± 1.76	0.71	4.29 ± 1.84	0.22
DASH	32.67 ± 14.73	31.35 ± 16.26	0.42	20.34 ± 11.76	< 0.05	12.58 ± 6.57	< 0.05
Kapandji	9.35 ± 1.42	8.95 ± 1.05	0.34	9.50 ± 0.76	0.69	9.55 ± 0.69	0.59
VAS	6.05 ± 2.31	3.75 ± 2.38	< 0.05	2.15 ± 1.46	< 0.05	1.10 ± 1.12	< 0.05
Mayo Wrist Scores	42.00 ± 12.07	55.00 ± 13.57	< 0.05	68.00 ± 8.34	< 0.05	74.75 ± 5.95	< 0.05
*Radiographic parameters*							
Radial abduction	27.33 ± 10.8	42.14 ± 8.48	< 0.05				
Palmar abduction	12.47 ± 7.40	34.36 ± 10.55	< 0.05

**Fig. 3:**
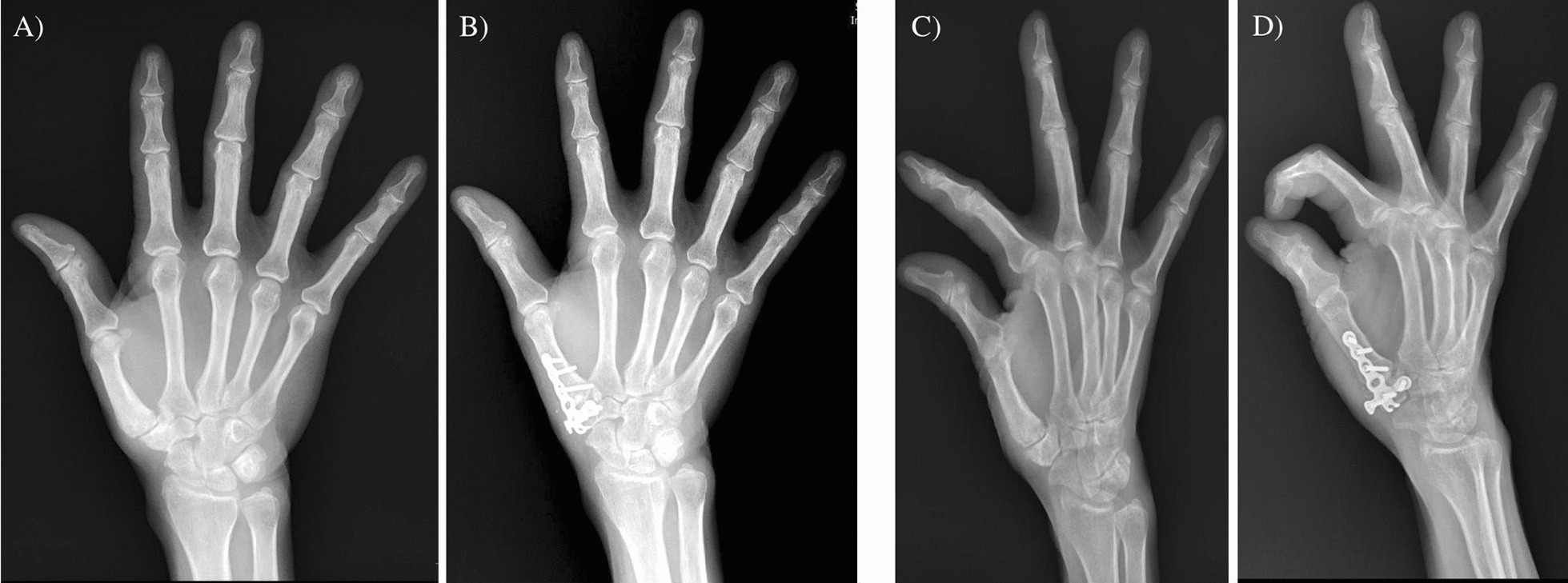
Hand X-rays **A** AP and **C** oblique preoperative X-rays; **B** AP and **D** oblique postoperative X-rays with T-hook plate CMC joint fusion

**Fig. 4:**
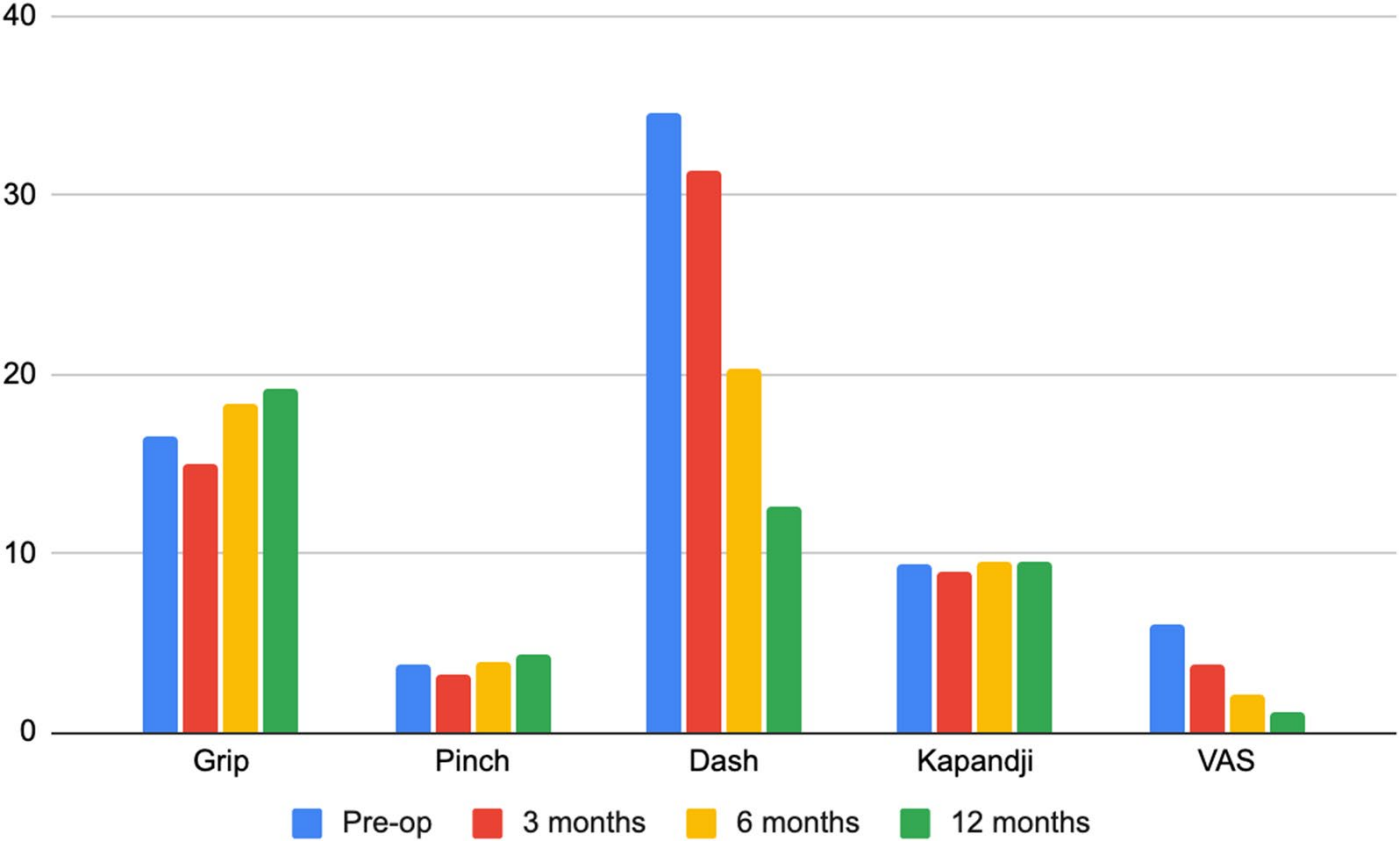
Preoperative and postoperative trend of grip, strength, DASH, Kapandji and VAS scores

Figure 3 showed preoperative and postoperative hand X-rays of a 40 years old female with Eaton stage III thumb CMCJ OA. Fusion was confirmed through X-rays; meanwhile, her pinching strength and capability of thumb opposition were restored after the fusion. In addition, she was found to have an improvement of 172% and 220% in her radial and palmar abduction angles, respectively. The preoperative and postoperative radial and palmar abduction angles of all patients evaluated on the hand X-rays were tabulated. Fusions within 6 months were reported in 100% of our cases and there were statistically significant postoperative improvements in both radial (25.33 ± 10.8 to 42.14 ± 8.48) and palmar abduction angles (12.47 ± 7.40 to 34.36 ± 10.55) (*p* < 0.05). Interestingly, our observations revealed a functional regression in grip, pinch, and Kapandji scores at 3 months post-operation. However, as seen in Fig. 4, improvements in all measured parameters were evident by 6 months of follow-ups, with all parameters surpassing preoperative levels at 12 months of follow-ups.

## Discussions

In our study, only Eaton III classification patients were included. Arthrodesis has been reported to maintain thumb length and pinch strength, however, it has not been widely accepted as the first-line treatment due to high incidences of loss of opponens function, which is a crucial function for human beings [16]. With advancements in osteosynthesis techniques in the recent decade, we can see that arthrodesis is gaining increased importance in the management of thumb CMCJ OA, particularly for Eaton III lesions. From our prior experiences, the bone quality of trapezium plays a critical role in determining successful postoperative fusion and union rates. Eaton IV usually involves degeneration of the STT joint and thus making trapezium unsuitable for arthrodesis. Regarding plate fixation technique, we choose T-hook plates (Acumed, USA) because its contour closely resembles that of the dorsal surface of the thumb CMCJ. The hook structure allowed firm fixation of the plate onto the trapezium with the body of the plate lying fit onto the thumb metacarpal bone. As seen from our operative technique, 3 proximal uni-cortical screws were used for trapezium fixation and the trajectory of the screws were placed in a convergence manner to allow stable fixation for successful fusion. Together with good trapezium quality, the structure of the T-hook plate was reliable to provide sufficient mechanical support and fixation strength for thumb CMCJ fusion. Our results further demonstrated the merits of this approach, which not only effectively alleviates pain but also preserves thumb strength by maintaining an appropriate joint length. Among the patient cohort we selected, no instances of non-union occurred, and 100% fusion rate was achieved with improvements in postoperative wrist functions. Furthermore, no bone grafts were required for all of our cases. After 12 months of postoperative follow-ups, there were no cases of infection or fractured plates. Only 1 case of radial nerve neuropathy was noted which was resolved within 6 months after implant removal with neurolysis.

The preferred position for fusion is 30– 40° palmar abduction and 10–20° radial abduction and extension [20]. This position is also described as “thumb key pinch”, where the thumb tip rests on the radial aspect of the middle phalanx of index finger. In our study, we would like to introduce a simple intraoperative hand positioning method that has been proven to provide convenience, replicability, and reliability of surgical results. By aligning the thumb tip with the PIPJ of the middle finger and placing the wrists in a clenched position throughout the surgery, we reported that no intraoperative adjustment of positions and additional intraoperative fluoroscopies were required except for confirmation of screw length and placement. Most importantly, our results showed that we were able to improve postoperative palmar and radial abduction angles and simultaneously preserving opponens function. Contrary to previous assumptions regarding potential loss of opponens function due to arthrodesis, our evaluation with Kapandji score did not reveal any significant functional deficits, thus indicating that our intraoperative positioning method was suitable for arthrodesis and maintaining postoperative opponens functions. This improvement could also potentially be attributed to the alleviation of pain, as evidenced by the pain VAS score results, thereby leading to increased willingness for finger movement among patients. Even though there were no statistically significant differences in pinch and grip strength data, postoperative follow-up data at 6 and 12 months showed gradual improvements in all measured parameters, even surpassing preoperative levels after 12 months of follow-ups. A decrease in functional performance was observed at 3 months postoperatively, which was similar to our previously reported results [21].

Overall, our findings showed positive results of arthrodesis in treating Eaton III thumb CMCJ OA patients, highlighting its benefits, while recognizing its associated drawbacks. Our study was a midterm follow-up study with 12 months of follow-ups. Future studies could involve a longer follow-up duration to better understand the recovery trend and to observe for complications such as secondary arthritis of the STT joints and other neighbouring joints. Nevertheless, our intraoperative positioning method not only offered convenience and easy replicability to hand surgeons, it was also shown to provide satisfactory midterm postoperative functional results; therefore, we confidently recommended our technique for thumb CMCJ fusion of Eaton III OA patients.

## Conclusions

Osteoarthritis of the thumb carpometacarpal joints are increasing due to our lifestyle and the trend of an ageing population. However, there are currently still no consensus regarding an optimal surgical treatment. Our retrospective review showed the reliable result of T-hook plate arthrodesis for Eaton stage III patients. Decreased pain, preserving opponens functions and pinch power were achievable, leading to improved quality of life for the patients. Therefore, we highly recommend our intraoperative hand-grip positioning method and T-hook plates for thumb carpometacarpal joint arthrodesis.

## Data Availability

The authors agreed for publication and data usage.
